# Advances in cartilage tissue regeneration: a review of stem cell therapies, tissue engineering, biomaterials, and clinical trials

**DOI:** 10.17179/excli2024-7088

**Published:** 2024-09-03

**Authors:** Julia Skoracka, Kaja Bajewska, Maciej Kulawik, Wiktoria Suchorska, Katarzyna Kulcenty

**Affiliations:** 1 Poznan University of Medical Sciences, Poznan, Poland, Fredry 10 Street, 61-701 Poznan, Poland; 2 Department of Electroradiology, Poznan University of Medical Sciences,Garbary 15 Street, 61-866 Poznan, Poland; 3 Radiobiology Laboratory, Greater Poland Cancer Centre, Garbary 15 Street, 61-866 Poznan, Poland

**Keywords:** cartilage regeneration, stem cells, tissue engineering, biomaterials, 3D bioprinting, clinical trials, chondrogenesis, extracellular matrix, exosomes, organ-on-a-chip

## Abstract

Cartilage tissue, characterized by its limited regenerative capacity, presents significant challenges in clinical therapy. Recent advancements in cartilage regeneration have focused on integrating stem cell therapies, tissue engineering strategies, and advanced modeling techniques to overcome existing limitations. Stem cells, particularly Mesenchymal Stem Cells (MSCs) and induced pluripotent stem cells (iPSCs), hold promise for cartilage repair due to their ability to differentiate into chondrocytes, the key cells responsible for cartilage formation. Tissue engineering approaches, including 3D models, organ-on-a-chip systems, and organoids, offer innovative methods to mimic natural tissue microenvironments and evaluate potential treatments. MSC-based techniques, such as cell sheet tissue engineering, address challenges associated with traditional therapies, including cell availability and culture difficulties. Furthermore, advancements in 3D bioprinting enable the fabrication of complex tissue structures, while organ-on-a-chip systems provide microfluidic platforms for disease modeling and physiological mimicry. Organoids serve as simplified models of organs, capturing some complexity and enabling the monitoring of pathophysiological aspects of cartilage diseases. This comprehensive review underscores the transformative potential of integrating stem cell therapies, tissue engineering strategies, and advanced modeling techniques to improve cartilage regeneration and pave the way for more effective clinical treatments.

## Integrating Cell Therapy and Tissue Engineering for Enhanced Treatment Strategies

### Utilization of stem cells

Stem cells have emerged as promising agents for tissue regeneration, particularly in cartilage repair. Their unique capacity to differentiate into diverse cell lineages, including chondrocytes, renders them highly valuable for replenishing damaged cartilage tissue. Mesenchymal stem cells (MSCs), sourced from tissues such as bone marrow or adipose tissue, have been extensively investigated for their chondrogenic potential. When appropriately guided, these MSCs can generate new cartilage matrix, offering a potential remedy for cartilage defects resulting from injury or degenerative conditions like osteoarthritis (OA). Moreover, induced pluripotent stem cells (iPSCs), derived from adult cells through reprogramming, present another avenue for generating chondrocytes for cartilage repair (Suchorska et al., 2017[28]; Augustyniak et al., 2017[1]). Exploiting the regenerative capabilities of stem cells holds significant promise for developing novel therapies aimed at restoring cartilage function and alleviating associated symptoms.

**Figure 1:**
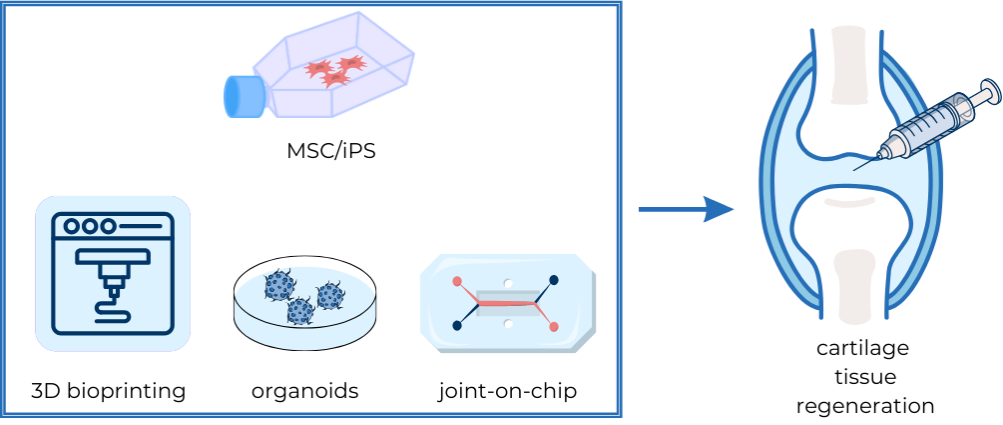
Stem cells and tissue engineering as an innovative approach to cartilage regeneration Stem cells, particularly mesenchymal stem cells (MSCs) and induced pluripotent stem cells (iPSCs), are key in cartilage regeneration for their differentiation into chondrocytes. 3D bioprinting, leveraging bioinks composed of cells and supportive materials, aims to create scaffolds that mimic the extracellular matrix, enhancing chondrocyte differentiation and tissue repair. Organoids, derived from pluripotent cells, offer simplified models of cartilage, enabling the study of diseases and therapeutic effects in a controlled environment. The joint-on-a-chip technology simulates cartilage conditions like osteoarthritis, incorporating fluid dynamics and synthetic components to model joint responses. Together, these approaches represent advanced strategies for studying and potentially healing damaged cartilage.

Stem cells (SCs) possess the remarkable capacity to differentiate into various cell types within the human body and exhibit a unique predisposition to self-renewal. These undifferentiated cells are categorized into different types based on their developmental potential and origin (Zakrzewski et al., 2019[41]). Certain types of stem cells hold particular promise in cartilage regeneration due to their ability to differentiate into chondrocytes, notably MSCs (mesenchymal stem cells) and iPSCs (induced pluripotent stem cells) (Suchorska et al., 2017[27]). By implementing appropriately selected protocols, stem cells can be a promising starting point for cartilage regeneration (Figure 1(Fig. 1)).

#### Mesenchymal stem cells

Mesenchymal stem cells can be isolated from several sources, including human mature tissues such as bone marrow, adipose tissue, skeletal muscle, skin, or structures of fetal origin such as amniotic fluid, umbilical cord, and fetal liver (Chen et al., 2008[5]). MSCs can produce diverse extracellular matrix (ECM) components, crucial for the optimal functioning of cartilage tissue. Yang et al. investigated the properties of MSC-derived ECM from human bone marrow (hBMSC-ECM) as a constituent of an *in vitro* chondrocyte culture medium. The cultured hBMSC-ECMs were harvested, with the living cells removed, yielding a decellularized extract. It was observed that cells cultured on hBMSC-ECM exhibited accelerated proliferation compared to the control group, which lacked this factor. Chondrocytes cultured on hBMSC-ECM maintained a more favorable phenotype specific to this cell type, as evidenced by a higher ratio of collagen type II to collagen type I gene expression and lower expression of collagen type X and ALP. High-density micromass culture in a chondrogenic medium with TGFB3 enabled the assessment of cell differentiation with and without ECM medium, revealing a significantly enhanced chondrogenic differentiation profile in the ECM group (Yang et al., 2018[40]). 

A plethora of growth factors play significant roles in cartilage regeneration. Given previous findings indicating the ability of basic fibroblast growth factor (bFGF) to promote cartilage proliferation and MSC differentiation *in vitro*, Okamura et al. investigated this phenomenon *in vivo* using a mouse model (Okamura et al., 2021[21]). Synovial mesenchymal stem cells (SMSCs) were cultured under two conditions: with or without bFGF in a growth medium. The SMSCs were aggregated, and the resulting synovial pellets were implanted into osteochondral defects in the femoral condyles of SCID mice. Histological assessment depicting cells stained with human vimentin confirmed the presence of administered SMSCs. Pronounced lacunar structures and cartilage substrate stained with safranin-O were observed only in the bFGF(+) group of mice, indicating superior cartilage regeneration following the addition of this growth factor.

Exosomes secreted from stem cells can also provide cartilage tissue regeneration support. Exosomes participate in numerous physiological and pathological processes and carry genetic information. Wang et al. demonstrated in a mouse model of DMM (destabilization of the medial meniscus) that exosomes from embryonic mesenchymal stem cells facilitate cartilage regeneration in osteoarthritis (OA) (Wang et al., 2017[37]). Injection of ESC-MSCs led to cartilage tissue regeneration, as confirmed by *in vitro* studies. Immunohistochemistry revealed that this effect was mediated by secreted exosomes, which enhanced collagen type II synthesis and reduced ADAMTS5 expression in the presence of IL-1B. Exosomes from ESC-MSCs exhibited a therapeutic effect on OA by balancing chondrocyte ECM synthesis and degradation. Another illustrative example of the potential of exosomes in cartilage tissue disease therapy stems from research was conducted by Tao and colleagues (2017[30]). Using a rat model, the research team investigated exosomes derived from synovial membrane-derived mesenchymal stem cells (SMSCs) overexpressing miR-140-5p (SMSC-140-Exos) in OA therapy. The results indicated that exosomes without miR-140-5p overexpression (SMSC-Exos) delivered Wnt5a and Wnt5b, stimulating YAP via an alternative Wnt signaling pathway, thereby increasing chondrocyte proliferation and migration. However, this led to reduced SOX9 expression and impaired secretion of ECM components essential for creating an appropriate environment for cartilage regeneration. Overexpression of miR-140-5p via SMSC-140-Exos mitigated these adverse effects by inhibiting RaIA and restoring proper SOX9 expression. It was confirmed that such exosomes facilitated substantial cell proliferation while maintaining appropriate ECM secretion *in vitro*, with *in vivo* studies on a rat model demonstrating a preventive effect against OA. 

Clinical trials also provide evidence of the efficacy of cartilage regeneration with mesenchymal stem cell therapies. The results raise hopes for using such treatment in clinical practice. Such an example is a prospective study conducted in Japan, the purpose of which was to compare alterations in the projected cartilage area ratio (thickness ≥ 1.5 mm) at the femoral posteromedial region in a time frame from 30 weeks before MSCs injection to 30 weeks after cell delivery, pointing out that cell injections were performed at the beginning of the study and again 15 weeks later. Patients with osteoarthritis who experienced knee discomfort and pain were recruited. The predicted cartilage area ratio decreased significantly by 0.07 in the 30 weeks up to the time of MSC injection, but there was no further decrease afterward. 3D MRI analysis showed that MSCs synovial injection slowed cartilage loss in the knees of treated OA patients. In addition, there was a significant increase in scores on scales relevant to the evaluation of OA, such as the Lysholm Knee Score, KOOS, and NRS (Sekiya et al., 2021[24]).

#### Induced pluripotent stem cells

Induced pluripotent stem cells (iPSCs) represent a class of stem cells derived artificially from non-pluripotent cells, such as human somatic cells, through the enforced expression of key genes characteristic of embryonic stem cells. This method enables the generation of cells exhibiting properties remarkably akin to naturally occurring pluripotent cells, including similar gene expression profiles, protein and receptor profiles, morphology, and differentiation potential (Takahashi and Yamanaka, 2006[29]). Thus, iPSCs hold considerable promise in the realm of cartilage regeneration. 

Lee and colleagues (2021[17]) conducted a study where human-induced pluripotent stem cells were differentiated into mesodermal and ectodermal lineages to produce and compare chondrocytes derived from mesodermal cells (MC-Chs) and neural crest cells (NCC-Chs). Both types of chondrocytes exhibited markers characteristic of hyaline cartilage. Remarkably, NCC-Chs demonstrated greater morphological and transcriptional resemblance to native joint chondrocytes. In a rat model, implants of NCC-Chs transfected with growth factors promoted articular cartilage regeneration more effectively than MC-Chs. 

Lach and colleagues (2018[14]) investigated how varying the number of cells in embryoid bodies (EBs) derived from human embryonic stem cells influences their chondrogenic differentiation potential, focusing on the effects of cell count on nutrient access, oxygen distribution, and cellular interactions. Results show that EBs formed with 500 cells per well exhibit the highest mesodermal and prochondrogenic properties, achieving more efficient differentiation into chondrocyte-like cells by day 5 compared to larger and older EBs. This highlights the importance of cell number and culture duration in optimizing cartilage regeneration strategies using pluripotent stem cells (Lach et al., 2018[14]). The same research group presented a novel approach to articular cartilage regeneration using iPSCs, focusing on serum- and feeder-cell-free differentiation protocols in chondrocyte-like cells using fetal bodies. It highlights the development of a strictly defined and controllable method tested in monolayer and 3D cultures, with the latter showing enhanced chondrogenic gene expression and specific extracellular matrix deposition. This study marks an important step in clinical use to achieve early-phase chondrocyte-like cell differentiation in a completely controlled environment without animal serum or feeder cells (Lach et al., 2019[16]).

Limraksasin and colleagues (2020[18]) demonstrated the generation of a hybrid bone/cartilage complex *in vitro* using iPSCs. Mouse iPSCs were cultured in a micro space environment to form 3D spheres. These iPSC spheres were subjected to culture conditions with osteogenic induction medium (Os induction) or chondrogenic induction medium (Os-Chon induction). Os induction led to robust mineralization and a small amount of cartilage-like tissue. In contrast, Os-Chon induction promoted a mesodermal lineage with elevated expression of the lateral plate and paraxial mesoderm marker genes. This study underscores the feasibility of generating hybrid osteochondral tissue from iPSCs, with the relative proportions of bone and cartilage modulated by selecting appropriate induction protocols. 

iPSCs serve as a promising source for generating mesenchymal stem cells (MSCs), which, as previously mentioned, can readily differentiate into chondrocytes. Chang and colleagues (2020[4]) evaluated the therapeutic potential of iPSC-mesenchymal stem cell-derived chondrocytes (iPSC-MSCs) in a rabbit model of osteoarthritis (OA). iPSCs were confirmed to express pluripotency markers (OCT4, SOX2, and NANOG), and an established line of iPSC-MSCs was obtained after 30 days of differentiation (Chang et al., 2020[4]). These iPSC-MSCs exhibited typical mesenchymal stem cell markers, including CD29, CD44, CD90, CD105, and HLA-ABC, and demonstrated successful differentiation into chondrocytes. The resulting cartilage exhibited lower IL-1β, TNF-α, and MMP13 expression levels than controls, highlighting the potential of iPSCs to yield appropriately differentiated MSC-chondrocytes capable of repairing cartilage defects.

### Tissue engineering: using materials to create 3D models/organs

The main characteristic of cartilage tissue is its low ability to regenerate independently. There are three main types of cartilage in the human body: hyaline cartilage (defects of this type of cartilage have the most significant impact on patients), fibrocartilage, and elastic cartilage (ICRS, 2023[12]). Methods like osteochondral allograft transplantation (allografts), mosaicplasty, marrow stimulation techniques, microfracture, autologous chondrocyte transplantation (ACT) - autografts that aim to harvest the cells and expand them *in vitro* culture, after which they are delivered under a periosteum flap to the defect site are still currently used in clinical therapy. The limitations of these methods mainly include the low number of cells acquired through biopsy (Zhang et al., 2009[42]; Chung and Burdick, 2008[6]).

Although they have positive results, there is still room for improvement in the availability of the tissue, possible complications concerning the donor sites, or induced immune response when it comes to allografts (Temenoff and Mikos, 2000[32]). That is where tissue engineering evokes such high hopes regarding possible future treatments for the damage caused by trauma or disease. The main goal that it strives for is to improve regeneration and repair of the injured cartilage. Numerous methods introduce tissue engineering to improve treatment. They mainly include 3D MSC (Mesenchymal Stem Cell) based techniques such as cell sheet tissue engineering (Thorp et al., 2021[34]), which this part of the review will focus on.

#### 3D models

Modern approaches to cartilage regeneration involve the utilization of Mesenchymal Stem Cells (MSCs), which possess the capacity for differentiation into chondrocyte phenotypes. While current strategies involve the delivery of patients' chondrocytes to the defect site, MSCs offer an advantageous alternative due to their ability to be isolated from various tissues, such as bone marrow or umbilical cord, overcoming the limitation of cell availability associated with traditional chondrocyte isolation (Thorp et al., 2021[34]). This approach addresses current clinical treatment limitations, including the scarcity of transplantable cartilage and the challenges associated with chondrocyte culture. Unlike chondrocytes, MSCs are readily bankable and culturable and exhibit chondrogenic differentiation potential. 

The capability of MSCs to be expanded *in vitro* and their theoretical accessibility make them an ideal source for chondrogenically differentiated three-dimensional constructs, offering the potential for enhanced treatment optimization and effective transplantation. However, MSCs have limitations, such as poor survivability (Somoza et al., 2014[26]). 

Three-dimensional cellular constructs hold greater promise than their two-dimensional counterparts due to enhanced cellular interactions, leading to increased chondrogenesis (Zhang et al., 2015[43]). Although several studies involving 3D bioprinting on animal models have been conducted recently, human clinical trials are yet to be realized (Thorp et al., 2021[34]). 

3D printing aims to replicate tissue structures utilizing computer-based technology. Bioink, the material used for printing, typically comprises cells and a selected material, primarily as a substitute for extracellular matrix (ECM) to support cell behavior. Depending on the physiochemical properties of the artificial ECM, a suitable polymer can be selected (Kahraman et al., 2022[13]). 

3D bioprinting employs support materials to enhance cell adhesion to address poor cell retention. Various scaffolds are utilized to improve MSC differentiation, with biomaterials ranging from synthetic to natural polymers, the latter of which can be functionalized to enhance biochemical properties (Gomez-Salazar et al., 2020[9]). Control over the form of the construct is crucial for regulating chondrogenesis (Tatman et al., 2015[31]). However, using biomaterials in 3D printing for scaffold fabrication poses challenges regarding biocompatibility (Zhang et al., 2009[42]). Modern approaches aim to develop 3D MSC structures that eliminate the need for support materials. Cell sheet tissue engineering offers a scaffold-free alternative that preserves cellular interactions and enhances cell adhesion to damaged tissue post-transplantation (Thorp et al., 2020[33]).

### Organ-on-a-chip and organoids

Organ-on-a-chip systems are designed to replicate tissue interactions and physiological states within a microfluidic chip. Their primary advantage lies in their ability to mimic natural tissue microenvironments and functions accurately. By replicating organ physiology, these systems offer enhanced reliability in disease modeling and provide a higher degree of control due to their small scale (Cao et al., 2023[3]). For instance, a joint-on-a-chip model can simulate biochemical interactions and replicate cartilage diseases such as osteoarthritis. This model incorporates fluidic integrations to improve the replication of specific diseases and physiological responses of joints. Components such as a synovial membrane and chondrocyte emulators are necessary for the functionality of a joint-on-a-chip system (Banh et al., 2022[2]; Paggi et al., 2022[23]). 

Organoids represent an alternative method for mimicking cellular interactions. These three-dimensional structures are simplified versions of specific organs, capturing essential functions. While organoids are not as complex as fully functional organs, they can emulate some aspects of their complexity. Generated from pluripotent cells, organoids can serve as monitoring devices for the pathophysiological aspects of cartilage diseases. They primarily mimic how certain therapeutic approaches function within a complete organ context (Lin et al., 2023[19]). 

### Limitations of stem cell based approaches

In addition to promising advantages and applications, stem cell-based approaches also have limitations that must be mentioned.

Several issues related to MSCs require attention. The invasive way they are procured and the limited ability to deliver MSCs in significant quantities while maintaining high quality are substantial challenges. The proliferative and differentiation potential of MSCs decreases with age and in patients with bone or metabolic diseases (Lach et al., 2022[15]).

Recent studies have shown that MSCs derived from different sources, such as bone marrow (BM-MSC), umbilical cord (UC-MSC), and adipose tissue (AT-MSC), possess unique paracrine and immunomodulatory qualities and contribute to the development of diseases. AT-MSCs and UC-MSCs showed higher procoagulant properties, which raises safety concerns (Wu et al., 2020[39]).

A further obstacle is the need for a standardized, well-established differentiation technique that complies with Good Manufacturing Practice (GMP) guidelines. Some protocols still rely on animal ingredients, such as fetal bovine serum, which may lead to variability in culture conditions, risk of zoonotic disease transmission, and ethical issues (Desai et al., 2015[7]).

One of the main limitations of using iPSCs is the method of obtaining them. Most methods for generating pluripotent stem cells use viral vectors such as retroviruses and lentiviruses that integrate randomly into the host cell genome. This may lead to genetic instability or disruption of the proper functioning of integrated genes, increasing the risk of cancer (Lach et al., 2022[15]; Omole and Fakoya, 2018[22]).

An important difference between MSCs and iPSCs is the ability to differentiate. MSCs can only differentiate into cells of the mesenchymal germ layer, whereas iPSCs can differentiate into cells from all three layers. This property of iPSCs poses a high risk of teratoma formation due to potential residual cells in newly formed tissue or organs (Gutierrez-Aranda et al., 2010[10]).

Another significant problem is the risk of rejection of an iPSCs-derived transplant. In the case of autologous transplantation, there is a risk of rejection, which may be the result of high cell immunogenicity, late passages of iPSCs cultures, or reprogramming methods using retroviral vectors (Garreta et al., 2018[8]).

The search for effective methods of treating osteoarthritis has aroused considerable clinical interest in differentiating iPSCs into chondrocytes of articular cartilage. However, current techniques are insufficient long-term and are mainly limited to younger patients with small lesions. Damaged human articular cartilage does not heal independently due to the high content of extracellular matrix and the lack of lymphatic vessels, vascular and nervous tissue (Lach et al., 2022[15]). 

Additional limitations include the need to develop safe, highly efficient protocols for differentiation into desired cells and problematic cost and logistic aspects associated with using iPSCs technology in regenerative medicine (Zimmermann et al., 2012[44]).

In conclusion, both iPSCs and MSCs have their unique challenges and limitations that must be overcome to be effectively used in articular cartilage regeneration. The development of safe, efficient, and cost-effective protocols is critical to the future success of these therapies in clinical practice.

## Clinical Trials in Cartilage Defects

The investigation presented in this article draws upon a comprehensive review of diverse clinical trials sourced from the *ClinicalTrials.gov* repository, thereby furnishing valuable insights into the realm of cartilage defects and stem cell-based interventions. Table 1(Tab. 1) (References in Table 1: Akershus, 2015[35]; Haleem et al., 2010[11]; Medipost Co. Ltd., 2021[20]; Solheim et al., 2016[25]; Volz et al., 2017[36]; Weiss, 2021[38]) delineates these trials, organized according to the classification of cartilage defects under the "condition or disease" category and stem cell-related interventions under "other terms" (accessed on May 31, 2023). A total of 19 trials were identified, with outcomes available for 18 among them. Subsequently, the ensuing section succinctly encapsulates the findings pertinent to cartilage regeneration, specifically emphasizing clinical outcomes. Notably, our focus was directed towards trials with published results.

### “Clinical Application of PRF Scaffold in Bone Marrow Stem Cell Transplantation for Cartilage Repair” (NCT00891501)

This pilot study investigated the clinical application of a platelet-rich fibrin (PRF) scaffold in conjunction with bone marrow-derived mesenchymal stem cell (BM-MSC) transplantation for cartilage repair in patients with cartilage lesions. The trial was conducted at Seoul St. Mary's Hospital, Republic of Korea, and commenced in April 2009, with completion in October 2013. In this study, conducted on 5 patients with cartilage lesions, a platelet-rich fibrin glue scaffold was used as a carrier for bone marrow-derived mesenchymal stem cells (BM-MSC). These autologous stem cells, cultured *in vitro*, were then applied to the glue plate and transplanted onto femoral cartilage. The results, evaluated 6 and 12 months post-surgery, demonstrated significant improvements in cartilage condition and partial or complete filling of damaged tissue. Notably, the study highlighted the efficacy of BM-MSC on a scaffold for treating cartilage defects and validated the utility of MRI as an effective postoperative assessment tool (Haleem et al., 2010[11]).

### “Evaluation of Re-Joint® for Knee Osteoarthritis” (NCT02855073), Phase IIa Clinical Trial

This study aimed to assess the safety and efficacy of Re-Joint®, a novel therapy consisting of autologous adipose-derived mesenchymal progenitor cells and sodium hyaluronate, in patients with knee osteoarthritis. The trial was sponsored by Chonnam National University Hospital, Republic of Korea, and commenced in July 2016, with completion in December 2019. The study enrolled patients diagnosed with knee osteoarthritis, a degenerative joint condition characterized by cartilage loss and inflammation. Participants were randomized into three groups based on treatment modalities, including microfracture surgery with different injections. The first group underwent microfracture surgery and an injection of sodium chloride solution; the second one was treated with a microfracture surgery and an injection of sodium hyaluronate; the third one underwent microfracture surgery, an injection of sodium hyaluronate, and the Re-Joint® transplantation. No serious side effects were observed in this study. In the first group, improvement was observed, but after a few months, the effects disappeared. In other groups, effectiveness decreased over time, but patients with Re-Joint® had the best results in some cartilage tests. This trial confirmed the safety and effectiveness of Re-Joint® therapy. While all groups showed varying degrees of improvement, the group receiving Re-Joint® demonstrated the most favorable results in certain cartilage tests, confirming the therapy's safety and efficacy (Weiss, 2021[38]). 

### "Long-term Outcome of Microfracture in Knee" (NCT01747681)

The primary focus of this study was to evaluate the long-term outcomes of microfracture surgery in patients with knee joint issues. The trial was sponsored by the Hallym University Medical Center, Republic of Korea, and commenced in December 2012, with completion in December 2022. While the trial's primary objective is not specifically centered on stem cell usage, microfracture surgery, the intervention under investigation, can indirectly involve activating stem cells within the bone marrow. Microfracture surgery is a minimally invasive procedure commonly used to treat minor cartilage defects in the knee. During the surgery, small holes are created in the subchondral bone, stimulating the release of bone marrow-derived stem cells into the defect site. These stem cells have the potential to differentiate into chondrocytes, the cells responsible for cartilage formation, and contribute to the repair of the cartilage defect. While the trial may not directly involve the administration of exogenous stem cells, the activation and recruitment of endogenous stem cells through microfracture surgery play a crucial role in the regenerative process observed in patients undergoing this procedure. Therefore, the trial may indirectly assess the effectiveness of stem cell-based approaches in cartilage regeneration by evaluating microfracture surgery outcomes over the long term. The study was performed on 110 patients. Post-operation patients were under the care of physiotherapists. Patients were followed up for 10 to 14 years post-surgery to assess the long-term efficacy of the procedure. This study was conducted 10-14 years later. Results show that the knee joint's normal function was never restored. Most of the patients required further operations to improve their quality of life. Nearly half of the subjects had poor results in cartilage tests (Solheim et al., 2016[25]).

### “Comparison of Microfracture Alone Versus Microfracture Combined With AMIC® for Cartilage Defects” (NCT02993510)

The study aimed to compare the efficacy of microfracture alone versus microfracture combined with AMIC® (Autologous Matrix-Induced Chondrogenesis) in patients with cartilage defects. AMIC® is a technique that involves the application of a collagen membrane to the defect site to enhance cartilage repair. The trial was sponsored by the University of Ulsan College of Medicine, Seoul, South Korea, and commenced in November 2016, with completion in January 2021. This trial conducted on 47 patients with cartilage defects compared the efficacy of microfracture alone versus microfracture combined with AMIC® (Autologous Matrix-Induced Chondrogenesis). Initially, the study started with 67 people. After 5 years, only 47 patients reached the endpoint because five centers that collected the data operated for only 2 years. In all cases, microfracture was used as a basic therapy. Patients were divided into three groups. The first group was treated only with microfracture, the second one underwent both microfractures and sutured AMIC®, and the third was treated with microfracture and glued AMIC® by fibrin glue. AMIC® connects treatment by microfracture with collagen type I/III matrix membrane called Chondro-Gide®. Results show that AMIC® improved the outcome of microfracture in cartilage defects. Both methods to fix Chondro-Gide® gave similar results. Side effects were not reported for all methods. Results demonstrated that AMIC® significantly improved the outcomes of microfracture in cartilage defects, with similar effectiveness observed between sutured and glued AMIC® methods and no reported side effects (Volz et al., 2017[36]).

### “Evaluation of the Safety and Efficacy of CARTISTEM® in Articular Cartilage Defects in the Knee” (NCT01733186)

This trial assessed the safety and efficacy of CARTISTEM® in patients with articular cartilage defects in the knee joint. Sponsored by Medipost Co., Ltd., South Korea, the trial commenced in November 2012 and was completed in February 2018. The CARTISTEM®, a cell therapy product derived from allogeneic umbilical cord blood-derived mesenchymal stem cells, was administered to participants via intra-articular injection. The efficacy of CARTISTEM® was evaluated in two study groups with varying sizes of cartilage defects. The first one was combined with patients with damaged cartilage size ranging from 2 to 5 cm^2^. Defects in the second group were above 5 cm^2^. The administrated dosage was 0.5 mL of the product per cm^2^. A total of 12 people participated in the study, 6 in each group. The study revealed that CARTISTEM® administration improved knee function, with better outcomes observed in patients with smaller defects. No severe side effects were reported, emphasizing the safety and potential efficacy of CARTISTEM® in treating cartilage defects (Medipost Co. Ltd., 2021[20]).

### "Cartilage Repair With Mesenchymal Stem Cells Derived From Nasal Turbinate in the Knee” (NCT00885729)

This study investigated the safety and efficacy of using mesenchymal stem cells (MSCs) derived from nasal turbinate tissue for cartilage repair in the knee joint. This study, sponsored by University Hospital, Akershus, Norway, was initiated in April 2009 and completed in June 2012. The study enrolled patients with knee cartilage defects who underwent arthroscopic surgery. MSCs derived from nasal turbinate tissue were isolated, cultured, and expanded *in vitro*. Subsequently, the cultured MSCs were implanted into the knee joint defect site using a fibrin glue carrier during arthroscopic surgery. The study's primary outcome measures included the procedure's safety, assessed by monitoring adverse events related to the MSC implantation, and the efficacy of cartilage repair, evaluated through imaging techniques such as magnetic resonance imaging (MRI) and clinical assessments of knee function. While specific results from the study are not provided in the summary, the completion of the trial suggests that the investigators were able to carry out the protocol as planned. Further details on the safety profile and efficacy outcomes of using MSCs derived from nasal turbinate tissue for cartilage repair in the knee joint will be available in the full study report or subsequent publications. In conclusion, clinical study NCT00885729 aimed to explore the potential of using MSCs derived from nasal turbinate tissue as a novel therapeutic approach for cartilage repair in knee joint defects, focusing on evaluating safety and efficacy outcomes (University Hospital, Akershus, 2015[35]).

## Notes

Julia Skoracka, Kaja Bajewska and Maciej Kulawik contributed equally as first author.

## Conflict of interest

Authors declare no conflict of interest.

## Figures and Tables

**Table 1:**
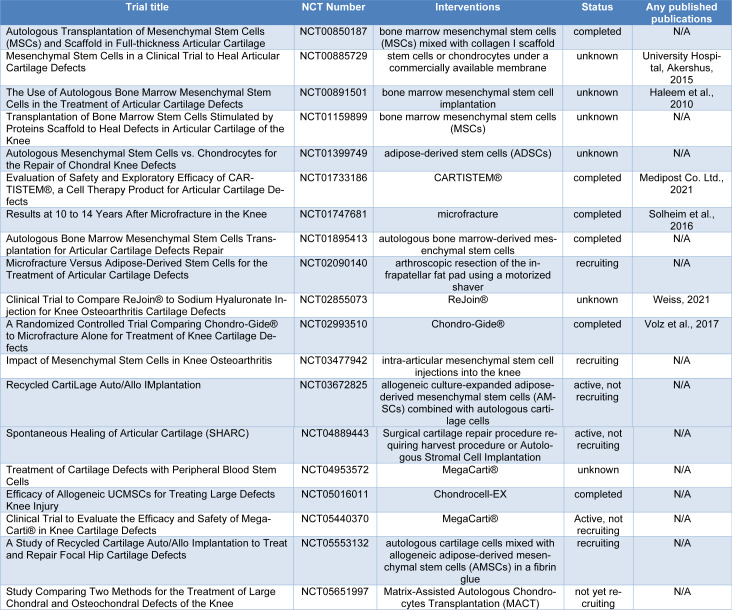
The described criteria searched the list of clinical trials (Clinicaltrials.com)
